# The Potential Role of Topical Losartan in Reducing Subepithelial Haze in Traumatic Laser-Assisted In Situ Keratomileusis (LASIK) Flap Avulsion

**DOI:** 10.7759/cureus.84337

**Published:** 2025-05-18

**Authors:** Stephen LoBue, Will Dietlein, Ayorinde Cooley, Thomas Lunsford, Curtis R Martin, Fatma Shakarchi, Nicolas Zaunbrecher, Wyche T Coleman

**Affiliations:** 1 Department of Ophthalmology, Willis-Knighton Medical Center, Shreveport, USA

**Keywords:** corneal haze, flap amputation, laser-assisted in situ keratomileusis (lasik), tgf beta, topical losartan

## Abstract

We report a case of a 42-year-old woman who previously underwent femtosecond-assisted myopic laser-assisted in situ keratomileusis (LASIK) with uncorrected distance visual acuity (UDVA) 20/20- in both eyes (OU). The patient presented three months later following a motor vehicle accident (MVA), which she experienced blunt force trauma from airbag contact to the left eye (OS). In the emergency department, the patient complained of pain, decreased visual acuity, excessive tearing, and edema of the left eyelids. Slit lamp examination was consistent with a complete traumatic LASIK flap amputation OS. A bandage contact lens was placed combined with moxifloxacin eye drops. The UDVA was 20/400 OS. The patient was continued on moxifloxacin four times a day (QID) and transitioned to Tobradex drops QID at day nine with improvement in UDVA to 20/30-2. At three weeks post-MVA, the patient’s visual acuity decreased to 20/100 secondary to significant corneal haze. Topical losartan 0.8 mg/mL was added six times per day along with fluorometholone two times a day (BID). Six weeks later, visual acuity was measured as 20/50-1 with improvement of central corneal haze. The patient was continued on losartan for four months with resolution of the corneal haze and improvement in UDVA to 20/30. Uncorrected visual acuity after LASIK flap amputation is generally favorable, especially if corneal haze can be prevented. Topical losartan has anti-fibrotic activity through inhibition of the transforming growth factor-beta (TGF-β) signaling pathway, and in conjunction with topical steroids, may be effective in improving corneal haze in patients with a traumatic LASIK flap amputation.

## Introduction

Femtosecond laser-assisted in situ keratomileusis (LASIK) is a common kerato-refractive procedure that corrects refractive errors by removing the corneal stroma under a laser made flap [1,2]. Although femtosecond LASIK complications are rare, there has been ample documentation among the literature. Complications may occur intraoperatively or postoperatively. Intraoperative complications range from incomplete flap creation due to suction loss, flap tear, decentered ablation, interface debris, and opaque bubble layer (OBL). The postoperative complications include flap striae or dislocation, residual refractive error, diffuse lamellar keratitis (DLK), microbial keratitis, epithelial ingrowth, refractive regression, corneal ectasia, dry eye disease, and flap amputation [1,2].

Among these complications, traumatic flap amputation or avulsion is a very rare postoperative complication but can occur days to years following LASIK surgery [3]. Flap amputation or avulsion may occur secondary to varying mechanisms of blunt force trauma, including motor vehicle accidents (MVAs), contact sports, or mixed martial arts [4-9]. However, secondary sequelae from the blunt forced injury may result in corneal scarring or haze, limiting the best-corrected visual acuity (BCVA).

Corneal haze (e.g., scarring or opacification) arises from an inflammatory response to injury with subsequent migration and differentiation of keratocytes into myofibroblasts as a function of wound repair [10]. An essential molecular regulator of the fibrotic response is the cytokine transforming growth factor-beta (TGF-β). During injury to the epithelial basement membrane (EBM) or Descemet's membrane (DM), TGF-β is activated in the corneal stroma to initiate and sustain keratocyte to myofibroblast differentiation and extracellular matrix deposition [11]. If EBM or DM repair is prolonged, persistent TGF-β signaling occurs. This sustained activation causes dysregulation of extracellular matrix deposition, establishing a pro-fibrotic state and the development of corneal haze.

Corneal haze can develop following surgical procedures, requiring medical interventions. Topical corticosteroids are often used to suppress inflammation and fibrosis by downregulating TGF-β-mediated events and reducing inflammation [12].

Recently, losartan, typically used for cardiovascular disease, has been proposed as an alternative treatment for corneal haze. Topical losartan has demonstrated its use in reducing pre-existing corneal scars and haze in animal studies [13-15] and human case reports [16,17]. Losartan is proposed to attenuate fibrosis by inhibiting the TGF-beta signaling pathway, thus preventing myofibroblast proliferation and promoting myofibroblast apoptosis [17,18].

While accumulating evidence suggests losartan is a potential treatment for fibrotic corneal changes, there is still a need to explore different clinical contexts where it can be applied. To provide more clinical data on treatment paradigms regarding post-surgical corneal fibrosis, we report a case of traumatic LASIK flap amputation, complicated by significant prolonged corneal haze, which was successfully treated with topical steroids and losartan eye drops.

## Case presentation

**Figure 1:**
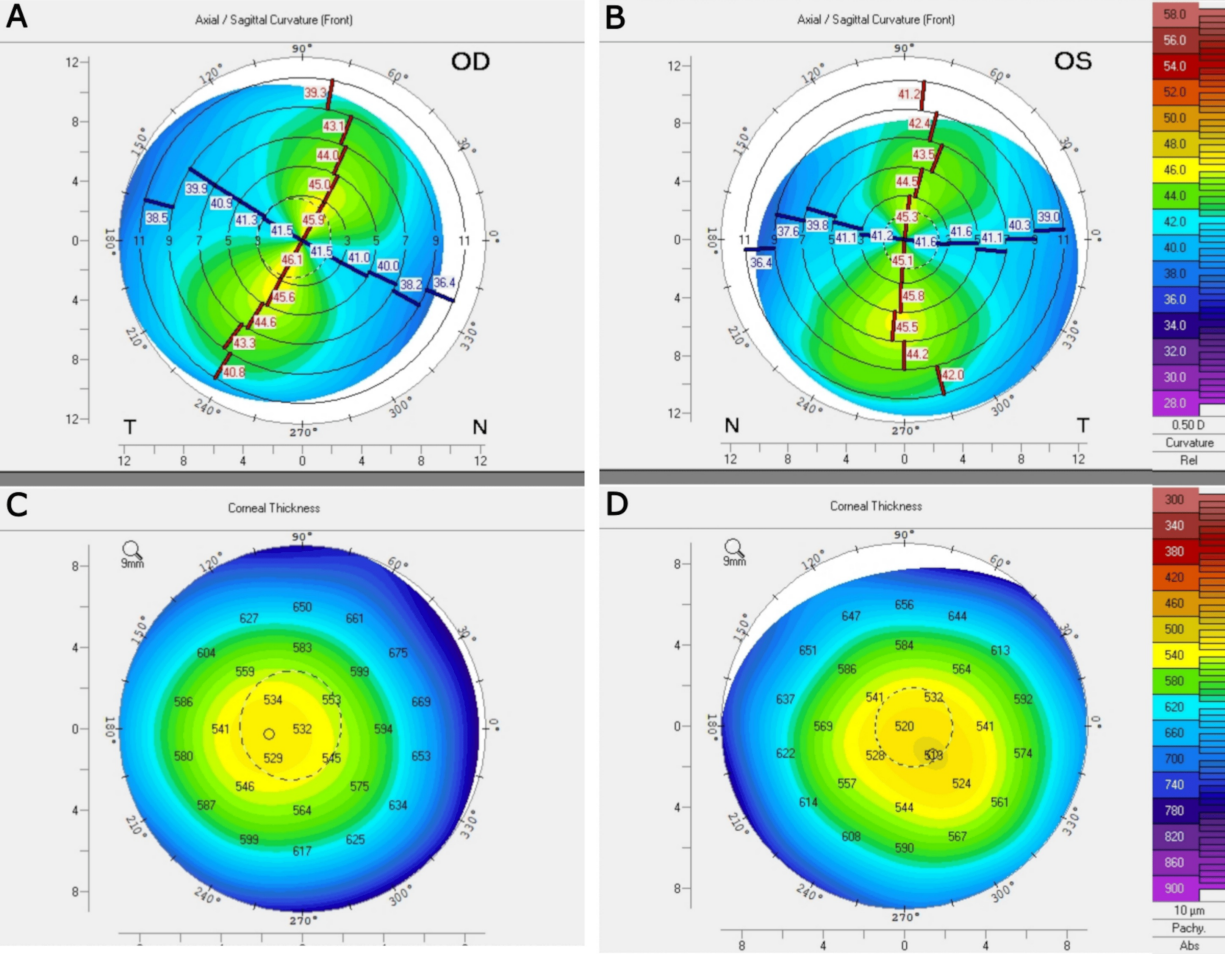
Preoperative topography demonstrating regular astigmatism (A) in the right eye (OD) and (B) regular astigmatism in the left eye (OS). Adequate central corneal thickness of (C) 526 μm OD and (D) 513 μm OS.

A 42-year-old woman with no past ocular or medical history presented for a refractive consultation. Manifest refraction was -4.75 +4.50 x 058 in the right eye (OD) and -4.50 +4.00 x 071 in the left eye (OS). Preoperative topography revealed regular astigmatism with adequate central corneal thickness (CCT) of 526 μm OD and 513 μm OS (Figure 1). The Belin/Ambrósio enhanced ectasia display demonstrated normal levels of anterior and posterior corneal elevation in both eyes (OU). No signs of underlying ectasia risks were observed in either eye.

Femtosecond-assisted LASIK surgery was performed, aiming for plano in both eyes. An 8.1 mm flap with a 100 μm thickness was created in both eyes using a VisuMax 500 femtosecond laser (Zeiss, Oberkochen, Germany). Wavefront-optimized ablation was performed after flap creation in both eyes (Wavelight Ex 500 Excimer Laser, Alcon, USA). The calculated residual stromal bed was 369 μm OD and 355 μm OS. The immediate postoperative period was uneventful. Six weeks postoperatively, the uncorrected visual acuity (UCVA) was 20/20-2 OD and 20/25+2 OS.

**Figure 2:**
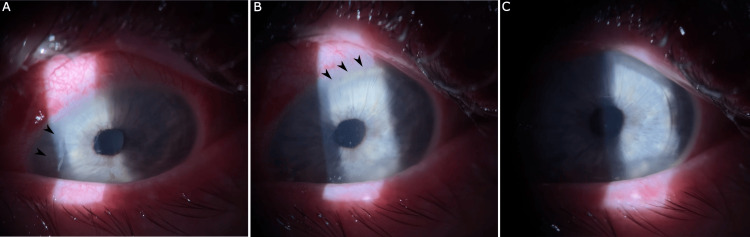
Slit lamp images following the motor vehicle accident (MVA). (A)-(C) Demonstrate loss of the corneal flap (black arrows) with surrounding conjunctival injection.

Postoperative month three, the patient experienced a significant motor vehicle accident (MVA) involving blunt force trauma from airbag deployment within her vehicle. Following the accident, the patient complained of pain, decreased visual acuity, excessive tearing, and lid edema OS. Visual acuity was measured at 20/20- OD and 20/400 OS. The intraocular pressure was 12 in both eyes (OU). On gross examination, edema of the left upper lid was observed, as well as a subconjunctival hemorrhage of the left eye. Slit lamp examination revealed findings consistent with traumatic LASIK flap amputation with complete loss of the corneal flap (Figure 2). A fundus exam was normal in both eyes, demonstrating no retinal hemorrhages, tears, or detachment. A bandage contact lens (BCL) was placed OS, and the patient was started on moxifloxacin eye drops QID.

**Figure 3:**
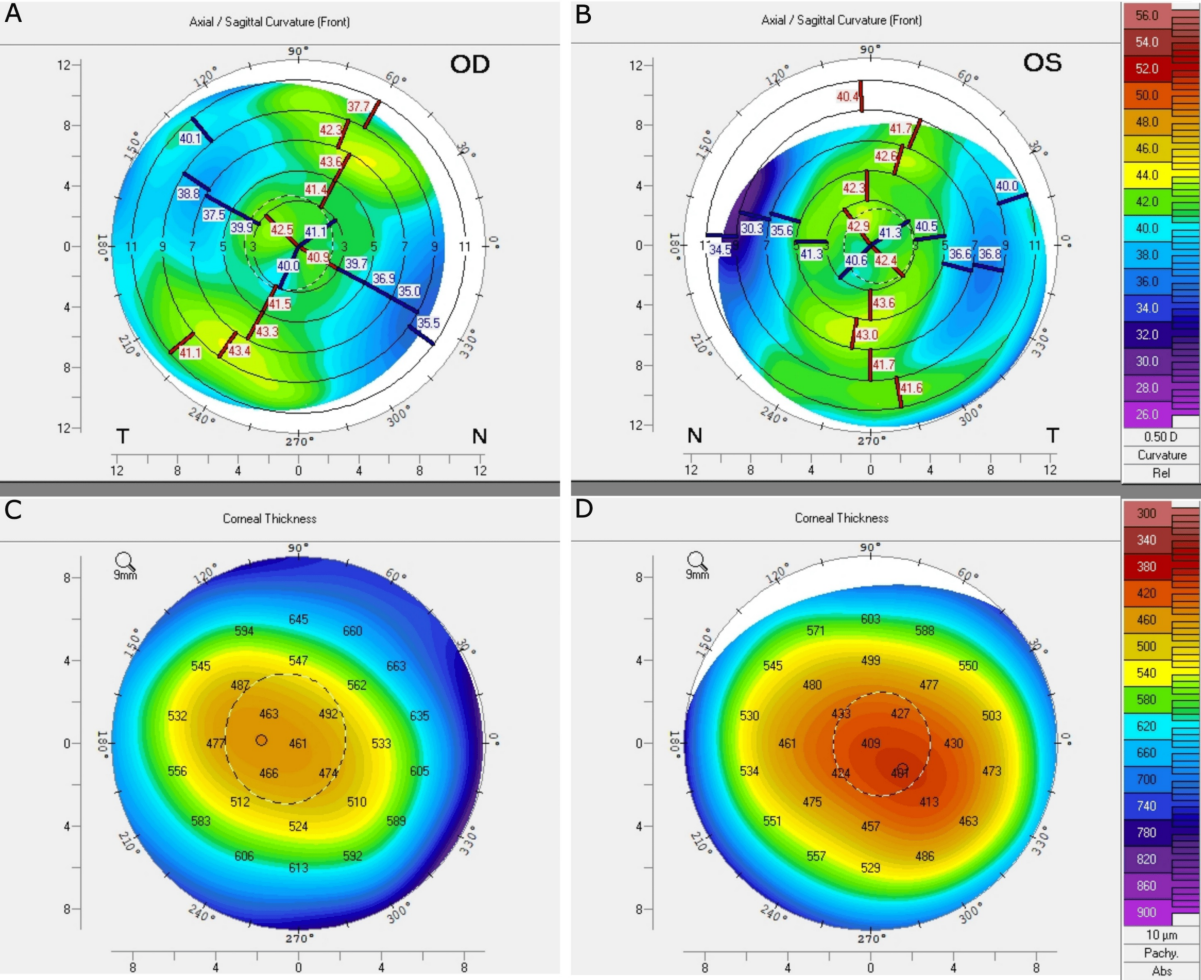
Repeat topography after the MVA, demonstrating minimal, regular astigmatism (A) in the right eye (OD) and (B) in the left eye (OS). Significant central corneal thinning from (C) post-surgery OD compared to (D) traumatic flap loss OS. MVA: motor vehicle accident.

**Figure 4:**
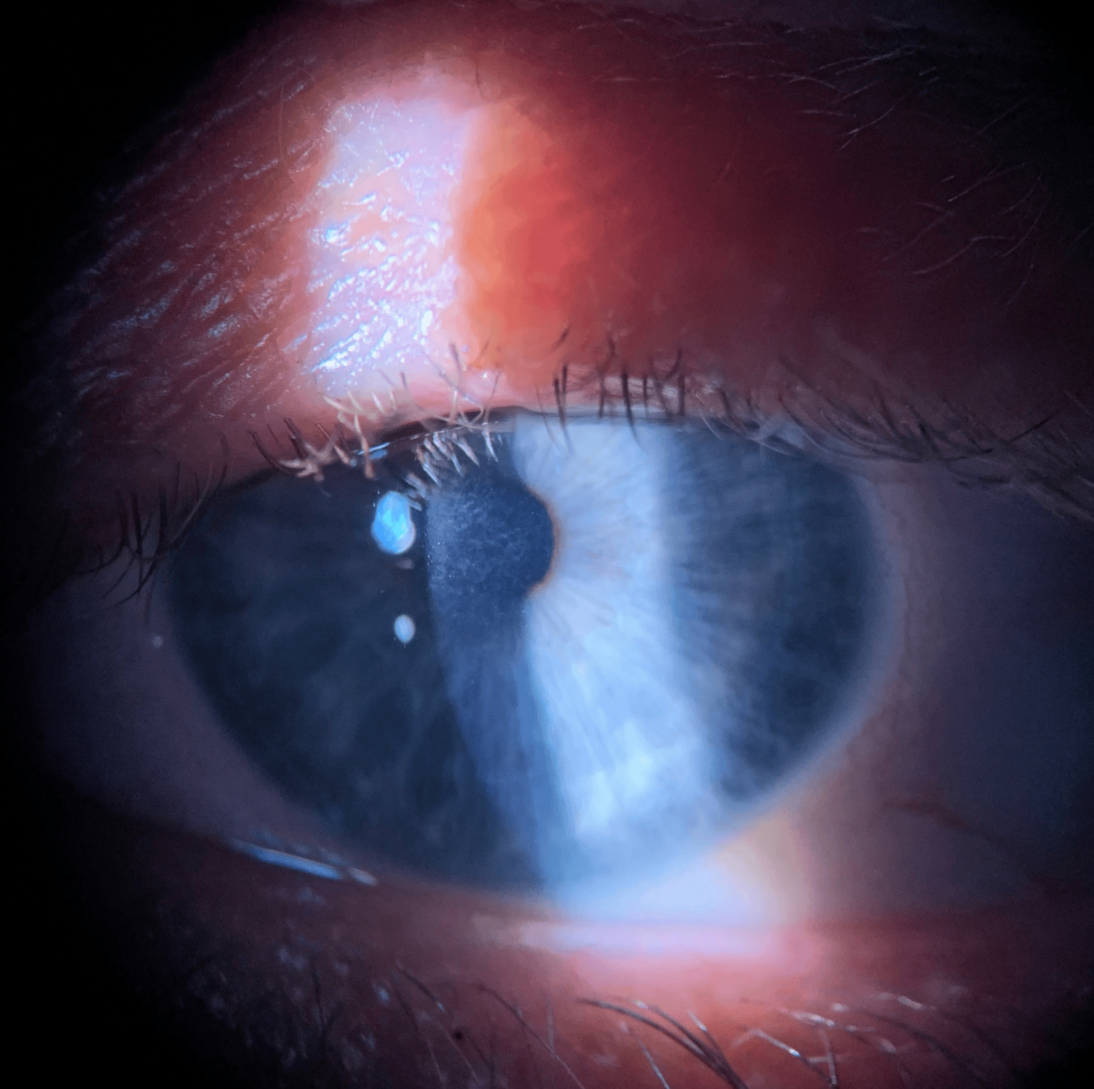
Follow-up on the 23rd day after the MVA revealed a 2x2 mm, central, subepithelial haze associated with a decrease in uncorrected visual acuity (UCVA) of 20/100. MVA: motor vehicle accident.

The patient was closely observed, with the removal of the BCL at one week post-MVA. At this point, the patient's uncorrected visual acuity was 20/30-2, and she was switched to Tobradex four times a day (QID) for a total of two weeks. Repeat topography demonstrated minimal, regular astigmatism in each eye with significant central corneal thinning OS > OD, consistent with flap loss (Figure 3). Follow-up on the 23rd day after the MVA revealed a 2x2 mm, central, subepithelial haze associated with a decrease in uncorrected visual acuity (UCVA) of 20/100 (Figure 4). In an effort to reduce and prevent any further corneal scarring, fluorometholone two times a day (BID) and topical losartan 0.8 mg/mL six times per day were initiated. Dexamethasone was not reinitiated in order to mitigate the risk of steroid-induced glaucoma as well as cataract formation in our patient. Topical losartan eye drops were created in a compounding pharmacy, utilizing a sterile technique.

**Figure 5:**
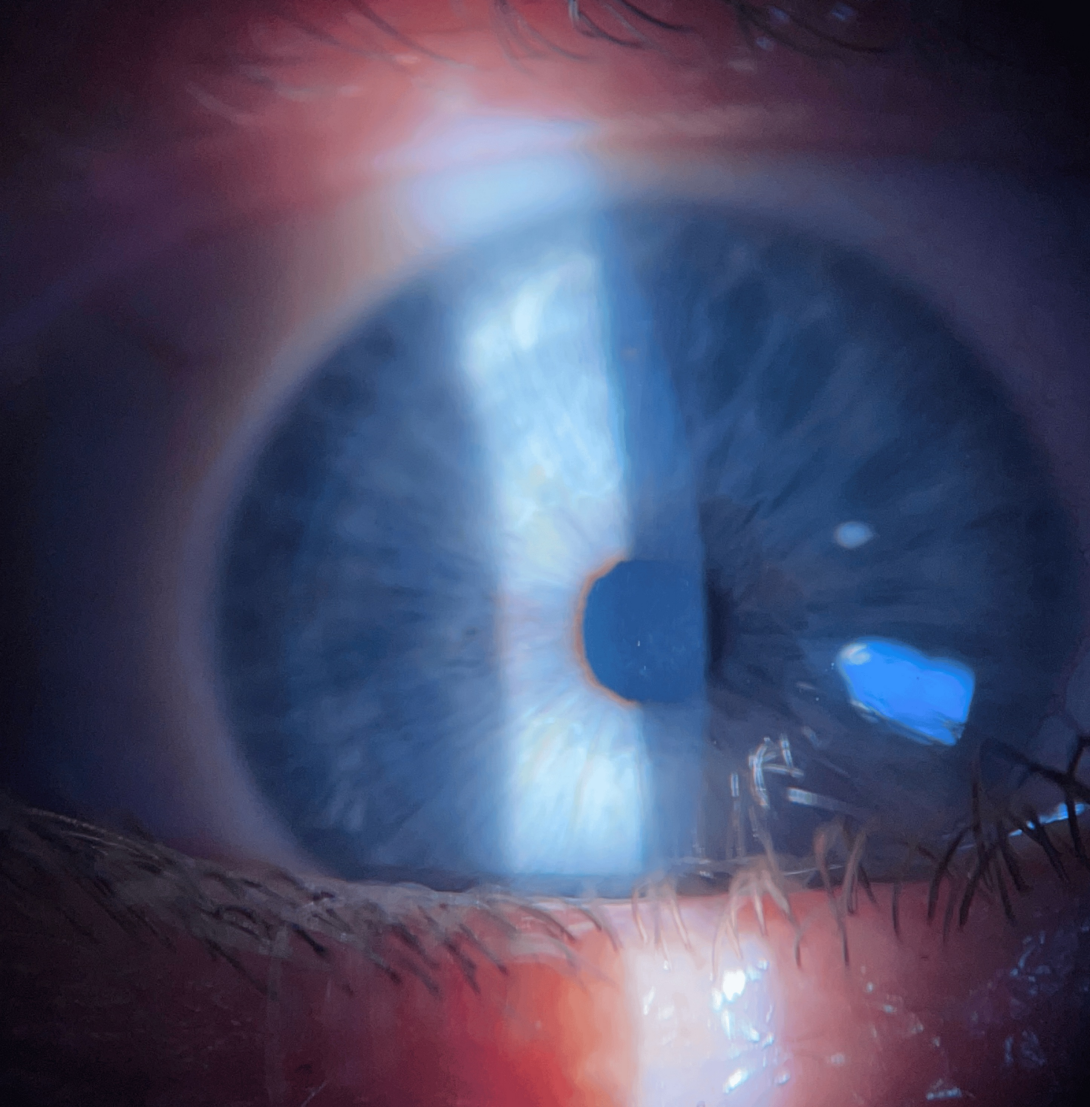
Significant reduction in the central corneal haze after four months of topical steroids and losartan eye drops.

Approximately two months after the MVA, visual acuity was measured as 20/50-1 with the central, 1x1 mm corneal haze. The patient was continued on losartan and fluorometholone for four months at the previous dosing. Four months after the MVA, the patient's uncorrected visual acuity was measured as 20/30-2 with a corrected visual acuity of 20/20-2. A complete reduction in the central corneal haze was noted at this time (Figure 5). Topical losartan and steroids were stopped, and the patient was scheduled for routine follow-up. No side effects from the topical drops were noted by the patient or the clinician. At this time, no further surgical intervention is planned as the patient is content with their vision.

## Discussion

Losartan is an angiotensin II (Ang II) receptor blocker (ARB) that interferes with TGF-β signaling by inhibiting activation of extracellular signal-regulated kinase (ERK) [19]. This interaction modulates the inflammatory response and reduces fibrosis by preventing the differentiation of fibroblasts into myofibroblasts and reducing extracellular matrix deposition. In the corneal stroma, TGF-β isoforms 1-3 can play different roles in the pro-fibrotic effects [20,21]. Furthermore, increased Ang II activity has been shown to inhibit corneal myofibroblast apoptosis, suggesting that blocking its cognate receptor through an ARB can reduce myofibroblast activity [22].

Several pre-clinical studies in rabbits have demonstrated that one month of 0.8mg/mL topical losartan was effective in reducing the fibrogenic marker, alpha-smooth muscle actin and stromal myofibroblast density, after deep corneal incision and phototherapeutic keratectomy (PTK), respectively [15]. In rabbits subject to photorefractive keratectomy (PRK), 0.2 mg/mL topical losartan reduced post-op corneal opacity and stromal myofibroblast density compared to controls [15]. In rabbits with alkali burn injuries, 0.2 mg/mL losartan was combined with 1% prednisolone acetate to decrease corneal haze one month after injury [13,14].

Topical losartan has been applied as a new adjuvant therapy to reduce corneal scarring in human studies. Our findings in this report are consistent with Pereira-Souza et al., who found that topical losartan 0.8 mg/mL six times per day effectively treated significant post-LASIK corneal haze that responded poorly to corticosteroid treatment [16]. After 4.5 months of topical losartan treatment, corneal haze was markedly reduced. Uncorrected and best-corrected distance visual acuity were improved from 20/200 to 20/30 and from 20/30 to 20/25, respectively. In another case, 0.8 mg/mL of losartan six times daily significantly reduced late-onset fibrosis in a radial keratotomy incision [17]. The same regimen of topical losartan was applied to reduce fibrosis following a contact lens-related corneal ulcer [17]. This treatment was also used in a patient who developed corneal haze following corneal crosslinking, with visual acuity improving from 20/150 to 20/40-1 after three months [16]. With our patient, we used low-dose corticosteroid treatment (loteprednol) concurrently with losartan to treat corneal haze. Topical dexamethasone was not utilized in order to mitigate the risk of steroid-induced glaucoma as well as cataract formation in our patient. As seen in other studies, losartan monotherapy was commonly implemented after failed reduction of corneal haze with corticosteroid treatments. However, our findings suggest that combining low-dose topical corticosteroids and topical losartan may be a viable treatment regimen to treat corneal haze.

Cases of significant subepithelial haze resulting in vision loss have been documented in cases of traumatic flap avulsion. In a study on outcomes of flap amputation after LASIK, a total of 67% (4/6 cases) had decreased BCVA secondary to subepithelial haze in patients with epithelial ingrowth undergoing flap amputation [23]. Table 1 demonstrates a summary of previously documented cases of complete LASIK flap avulsion. Among all traumatic flap avulsions from blunt trauma in Table 1, 50% developed corneal haze with decreased BCVA. Among surgeons, time since flap creation has always been correlated with flap strength. A prospective study utilizing a motorized pulling device examined normal eyes versus eyes with LASIK flaps, demonstrating that flap margin strength increases with time and has been documented to reach a maximum strength of 28.1% around 3.5 years compared to control corneal tissue [24]. Thus, flap dislocation later in the postoperative period will require higher levels of trauma and may be associated with a greater incidence of subepithelial fibrosis. 

**Table 1:** A summary of previously documented cases of complete LASIK flap avulsion with mean uncorrected distance visual acuity (UCDVA) and best-corrected distance visual acuity (BCDVA) after treatment with conservative therapies. BCDVA: best-corrected distance visual acuity; BCL: bandage contact lens; gtts: eye drops; FML: fluorometholone; MMC: mitomycin C; PTK: phototherapeutic keratectomy; UCDVA: uncorrected distance visual acuity; *: limited best-corrected visual acuity secondary to corneal haze; NA: not available.

Year	Author	Mechanism	N	Mean time to flap removal	Treatment	Mean UCDVA	Mean BCDVA
2021	Shih et al. [25]	Blunt trauma (basketball)	1	Four years	Surgical replacement	NA	20/20
2019	Galvis et al. [5]	Blunt trauma (wood handle)	1	13 years	FML 0.1%, gatifloxacin 0.3%, sodium hyaluronate 0.4%, gtts	20/100	20/30*
2011	Motwani et al. [6]	Blunt trauma (shovel)	1	Four years	PRK w/ MMC, FML, gtts	20/15	20/15
2009	Thomas et al. [9]	Blunt trauma (finger)	1	Two months	MMC 0.01%, BCL, gatifloxacin, prednisolone, gtts	20/20-2	20/20-
2007	Tetz et al. [8]	Blunt trauma (finger)	1	3.5 years	BCL, dexamethasone gtts	NA	20/20
2002	Eggink et al. [4]	Intraoperative free cap	2	Five days	Chloramphenicol 0.5%, FML, gtts	20/40-2	20/25*
		Extreme blinking	1	One day	Patching, PTK one month postoperatively	20/125	20/50*
2002	Sridhar et al. [26]	Blunt trauma (finger)	1	10 days	Patching, BCL	NA	20/70*

LASIK flap amputation may also be intentional in order to treat a variety of pathologies, including treatment-resistant infectious keratitis, epithelial ingrowth, diffuse lamellar keratitis, and astigmatism secondary to fixed flap striae (Table 2). Regardless of the underlying diagnosis, amputation of the flap is largely considered the last option in all cases that are resistant to all previous forms of treatment. Among these listed above, the most common indications for flap amputation are infectious keratitis and epithelial ingrowth [23].

Visual outcome following therapeutic flap amputation was reported by Castillejo Becerra et al. for 15 patients with a median final BCVA of 20/25 (range 20/20-20/200) [23]. Flap amputations for infectious vs non-infectious etiologies had worse baseline median uncorrected visual acuity (hand motion vs. 20/63, P < 0.001), an increase in surgical intervention (50% vs. 11%), and had worse final median BCVA (20/50 vs. 20/20, P=0.018), respectively (Table 2) [23]. Cases related to infection had a more guarded visual outcome secondary to corneal haze compared to non-infectious etiologies such as epithelial ingrowth [23]. 

For most cases, conservative management is adequate after complete amputation of the LASIK flap. Among all cases analyzed, only 11% (4/35 cases) required surgical intervention with a corneal transplant (Table 2). Depending on the etiology, management often includes agents to promote re-epithelialization of the cornea, treat or prevent infection, decrease inflammation, and reduce corneal haze. All therapies have the same goal: to prevent further damage and scarring of the cornea.

**Table 2:** A summary of documented cases of iatrogenic LASIK flap amputation due to various corneal pathologies and their associated mean UCDVA and BCDVA. BCDVA: best-corrected distance visual acuity; NA: not available; MMC: mitomycin C; TransPRK: transepithelial photorefractive keratectomy; UCDVA: uncorrected distance visual acuity; *: limited best-corrected visual acuity secondary to corneal haze; PK: penetrating keratoplasty.

Year	Author	Etiology	N	Mean time to flap removal	Corneal intervention	Mean UCDVA	Mean BCDVA
2024	Marques et al. [27]	Epithelial ingrowth	1	Five years	Flap amputation, MMC 0.02%	20/20	20/20
					Topography-guided TransPRK		
2023	Castillejo Becerra et al. [23]	Epithelial ingrowth	6	10.7 years	Flap amputation, lamellar keratoplasty (N=1)	20/40-	20/25*
		Infectious keratitis	6	Six years	Flap amputation, PK (N=3)	20/150	20/70*
		Diffuse lamellar keratitis	1	20 years	Flap amputation	20/150	20/20
		Vegetative foreign body	1	Seven years	Flap amputation	20/60	20/30
		Fixed flap striae	1	20 years	Flap amputation	20/30	20/25
2017	Chhadva et al. [28]	Epithelial ingrowth	2	NA	Flap amputation	20/30	NA
		Infectious keratitis	6	Two months	Flap amputation	20/50	NA
2002	McLeod et al. [29]	Epithelial ingrowth	2	Two months	Flap amputation	20/30	20/25+2
2000	Chung et al. [30]	Infectious keratitis	1	One month	Flap amputation	20/50	NA

Re-epithelialization of the cornea is completed with frequent lubrication with or without a bandaged contact lens (BCL). BCLs are typically avoided in active keratitis but can be an excellent adjuvant therapy to improve healing time and patient comfort in non-infectious etiologies. Topical ointment with or without antibiotic coverage can also be effective. Topical antibiotics, specifically fluoroquinolones, are also utilized to treat or prevent infectious keratitis after flap amputation. Our literature review revealed cases where gatifloxacin, moxifloxacin, and ofloxacin were commonly selected [5,9].

After complete flap amputation, reducing inflammation is achieved by topical steroids such as fluorometholone, prednisolone, and dexamethasone. Topical steroids should be incorporated once re-epithelialization of the cornea begins or after keratitis responds to treatment. Earlier addition of topical steroids is important to prevent or mitigate increased corneal scarring from infection or trauma. Vitamin C and Mitomycin C have also been used to disrupt corneal fibrosis after LASIK flap loss [8,9].

In our case, a BCL and topical antibiotics were initiated to promote re-epithelialization of the cornea while mitigating secondary keratitis. The early addition of strong topical steroids, such as dexamethasone drops, was not adequate to prevent corneal haze from developing. However, utilizing low-dose topical steroids (e.g., loteprednol) and topical losartan cleared visually significant corneal haze and improved the UCVA from 20/100 to 20/30-2 over four months. In our clinical experience, topical losartan with loteprednol may be an effective treatment to improve corneal haze after traumatic LASIK flap amputation.

Although we had success with topical losartan without any adverse effects, severe ocular toxicity has been document in rabbit models utilizing concentrations above 0.8 mg/mL [31]. Therefore, future studies on using losartan for corneal repair should focus on conducting large-scale randomized controlled trials to evaluate its safety and efficacy at differing concentrations as well as various corneal pathologies. Its effects should be observed through monotherapy and combined therapy with antibiotics and corticosteroids. Future clinical trials should also focus on developing optimized formulations and treatment schedules.

## Conclusions

LASIK flap amputation can result from severe blunt force trauma or can be utilized as a treatment for refractory infectious keratitis, epithelial ingrowth, DLK, and irregular astigmatism secondary to fixed flap striae. Depending on the etiology, management often includes agents to promote re-epithelialization of the cornea, treat or prevent infection, decrease inflammation, and reduce corneal haze. Uncorrected visual acuity after LASIK flap amputation is generally favorable, especially if corneal haze can be minimized. Topical steroids, along with topical losartan, may be beneficial in treating and preventing corneal fibrosis, which is the most significant cause of permanent vision loss following amputation. Overall, more clinical evidence is still required to elucidate the viability of topical losartan in treating and preventing corneal haze, as our data is limited to one case.
